# Description and Assessment of a Neurosurgery Shadowing and Research Program: A Paradigm for Early and Sustained Exposure to Academic Neurosurgery

**DOI:** 10.1515/tnsci-2019-0034

**Published:** 2019-08-09

**Authors:** Phan Q. Duy, Serban Negoita, Uma V. Mahajan, Nicholas S. Diab, Ank A. Agarwal, Trisha Gupte, Manish D. Paranjpe, William S. Anderson

**Affiliations:** 1 Department of Neurosurgery, Johns Hopkins University School of Medicine, Baltimore, MD, USA; 2 University of Maryland School of Medicine, Baltimore, MD, USA; 3 Case Western Reserve University School of Medicine, Cleveland, OH, USA; 4 Krieger School of Arts & Sciences, Johns Hopkins University, Baltimore, MD, USA; 5 Medical Scientist Training Program, Yale University School of Medicine, New Haven, CT, USA; 6 Department of Neurosurgery, Yale University School of Medicine, New Haven, CT, USA; 7 Harvard-MIT Program in Health Sciences and Technology, Harvard Medical School, Boston, MA, USA

**Keywords:** neurosurgery, medical education, pre-medicine, shadowing, research, surgical education

## Abstract

**Objective:**

To describe and assess the educational value of a functional neurosurgery clinical shadowing and research tutorial for pre-medical trainees.

**Design:**

Program participants observed functional neurosurgery procedures and conducted basic science and clinical research in neurosurgery fields. Former participants completed a brief online survey to evaluate their perspectives and experiences throughout the tutorial.

**Setting:**

Department of Neurosurgery, Johns Hopkins University School of Medicine, Baltimore, MD, USA.

**Participants:**

15 pre-medical and post-baccalaureate trainees participated in the tutorial. All former tutorial participants were emailed.

**Results:**

11/15 former participants responded to the survey. Survey results suggest that the tutorial program increased participants’ understanding of and interest in neurosurgery and related fields in neuroscience.

**Conclusions:**

The functional neurosurgery medical tutorial provides valuable clinical and research exposure in neurosurgery fields for pre-medical trainees. Our work is a preliminary step in addressing the crucial challenge of training the next generation of neurosurgeon-scientists by providing a pedagogical paradigm for development of formal experiences that integrate original scientific research with clinical neurosurgery exposure.

## Introduction

Predicated by its clinically relevant nature, translational research has long been amenable to surgeon scientists pursuing novel interventions or understanding of pathology. However the number surgeon scientists, as well as their relative funding from the NIH, has been decreasing [1]. This is despite continued emphasis by surgery departments on selection of residents, fellows and faculty with substantial research experiences [2]. A national survey of surgeons regarding perceived barriers in conducting basic and translation research identified increased clinical demands as the leading obstacle for surgeon scientists [1]. This finding may be particularly relevant to neurosurgery where regional and global shortages in the surgeon work force, along with deficits in emergency coverage present substantial burdens for practicing neurosurgeons [3].

In response to this challenging climate, some have argued that surgical programs must identify and support surgical trainees with a self-driven inclination for scientific investigation [4]. Cultivation of such surgeon scientists will require integrated exposure to clinical and research endeavors at different stages of training. Mentoring programs, which are crucial to developing the next generation of surgeons and scientists [5], have already been utilized to increase interest in particular clinical fields. A previously instituted shadowing program for trauma surgery at Johns Hopkins Hospital was successful at significantly increasing interest in trauma surgery for students not previously planning a career in trauma surgery, as well as increasing the percent of students planning to match into a surgical specialty [6]. Other shadowing programs, including cardiothoracic surgery and general surgery, have also been successful at increasing student interest and number of students applying to surgical residencies [7, 8].

These programs support that engaging trainees early their career is likely to have a significant impact on their selection of clinical specialties to pursue, and several formal programs currently exist to provide undergraduates or preclinical medical students exposure to neurosurgery [9, 10, 11]. In the US several summer programs are also available for pre-medical trainees to shadow and conduct research in neurosurgery [12, 13, 14]. These programs have been successful at increasing students’ confidence to engage with a neurosurgery department, increasing students’ likelihood to consider neurosurgery as a future career, and increasing positive perceptions of the associated quality of life for practicing neurosurgeons [15]. However, most programs that provide exposure to the neurosurgery field typically focus either on clinical experience or laboratory research; there currently exist few formal programs that simultaneously integrate both basic science research with clinical experience in the operating room [16]. Thus, most trainees receive very little exposure into the unique challenges and rewards of

being a clinician-scientist who conducts bench-to-bedside research, especially in clinically demanding clinical specialties such as neurosurgery. Providing trainees with integrative clinical and research exposure early in their careers will likely increase interest in pursuing academic medicine and inspire more trainees to become future neurosurgeon-scientists. Attracting the best students into neurosurgery is of paramount importance for the field, especially given concerns about the shortage of neurosurgeons in the US [17, 18, 19]. Here, we provide a description and assessment of a functional neurosurgery medical tutorial program that integrates clinical shadowing in the neurosurgery operating room with original scientific research activities for pre-medical trainees at the Johns Hopkins University.

## Description of program and goals

The functional neurosurgery medical tutorial (supported by both the Johns Hopkins University School of Medicine and the Johns Hopkins Homewood Pre-Professional Office) is a clinical shadowing and research program that allows undergraduate students at Johns Hopkins to shadow and conduct neuroscience research with Dr. William Anderson, a functional neurosurgeon at the Johns Hopkins Hospital. This tutorial is one of several medical tutorials offered in conjunction by the Johns Hopkins School of Medicine and the Homewood Pre-Professional Office, in which undergraduate students receive 1-2 academic credits graded on pass/fail basis [20]. These tutorials are offered in a number of areas including research (clinical, laboratory, community-based, or educational), hospital quality improvement, or practicum (shadowing).

The functional neurosurgery tutorial had two major goals: 1) Provide students with a clinical exposure to functional exposure and 2) Engage students in academic research in neurosurgery and related fields. Students participating in the functional neurosurgery tutorial were exposed to patients with epilepsy, movement disorders, and psychiatric conditions undergoing a variety of surgical procedures to help treat their conditions. Students participated in clinic, helping with patient flow and diagnostic study retrieval. They observed functional neurosurgery procedures, including those related to spinal cord stimulation for treatment of chronic low back pain, deep brain stimulation for Parkinson’s disease, and other therapeutic neuromodulation approaches using vagal nerve and cortical stimulation for epilepsy. To supplement their shadowing experiences, students were also able to attend a variety of clinical conferences and didactic activities in the hospital, including epilepsy case conferences and neurosurgery grand rounds. Based on interests, students had opportunities to contribute to research projects in the Anderson lab, including: 1) Understanding the neural network mechanisms of cognitive function, 2) Understanding the biological mechanism of neuromodulation therapies, and 3) Characterizing the clinical outcomes of functional neurosurgery procedures. Students have been co-author and first-authors on original research manuscripts as well as educational articles such as reviews in the field of neuroscience and neurosurgery [21, 22, 23, 24, 25]. These research experiences allow students to learn how to read academic literature, develop scientific questions and hypotheses, design experiments, perform retrospective chart reviews, analyze data, writing papers, and moving manuscripts through the peer-review process.

Students were required to meet two goals to obtain academic credit from the functional neurosurgery tutorial: come to at least one of the activities listed on the weekly functional neurosurgery schedule per week (this may include clinic, a surgical case, or a case conference); and meet with faculty member to review possible future research goals, and if interested participate in laboratory research. Students were graded based on degree of attendance- if the student made it to at least one scheduled event every other week, it would be a pass.

## Feedback from program participants

Out of a total 15 participants, 66.7% were female (Table 1). 46.7% were junior year students, 33.3% were sophomores, and 13.3% were post-baccalaureates. The most common major was neuroscience (46.15%), followed by biomedical engineering (23.1%) (Table). To assess the potential impact of the program, we administered an online anonymous survey to previous students (with approval from the Hopkins IRB, JHU IRB00165178). 11/15 (73%) of participants responded to the survey, and the results are shown in the Figure. The tutorial program appears to achieve its goals of exposing students and increasing interest in medicine, neuroscience research, and

**Table 1:** Demographics of participants in the functional neurosurgery tutorial. *The undergraduate major for two of the participants were not known

Fall 2013 – Spring 2016		n=15 participants
Gender	n	% of participants
Male	5	33.33
Female	10	66.67
Class year		
Sophomore	5	33.33
Junior	7	46.67
Senior	0	0
Postbac Pre-Med Program	2	13.33
Undergraduate majors*		
Neuroscience	6	46.15
Biomedical Engineering	3	23.08
Molecular & Cellular Biology	1	7.69
Chemistry	1	7.69
Public Health	2	15.38

the neurosurgery field. On a ten point scale (10 = strongly agree, 1 = strongly disagree), students rated a score of 8.5 ± 1.5 (mean ± SD) to the question “Participating in the functional neurosurgery tutorial allowed me to better understand what a future career in neurosurgery entails?” (Figure). To the question “The functional neurosurgery tutorial increased my interests in pursuing a future career in the neuroscience field”, students rated a score of 8.3 ± 1.7 (Figure).

## Discussion

Academic physician-scientists with dual training in basic science and clinical medicine are at the forefront of bench-to-bedside research to translate basic science discoveries of disease mechanisms into novel therapeutic avenues that improve patient outcomes. There is a profound clinical need for neurosurgeons who conduct original scientific research, as there is often a lack of satisfactory treatment strategies for neurosurgical disorders due to incomplete understanding into the pathogenesis of disease. Despite the great public need for academic surgeon-scientists, recent trends indicate alarming deficiencies in surgical research, including decreasing surgeon participation in basic science research [1]. Furthermore, surgeons are less likely to apply for NIH funding, and those who do tended to be less successful than their nonsurgical peers [26]. These trends have led to concerns about

**Figure 1:**
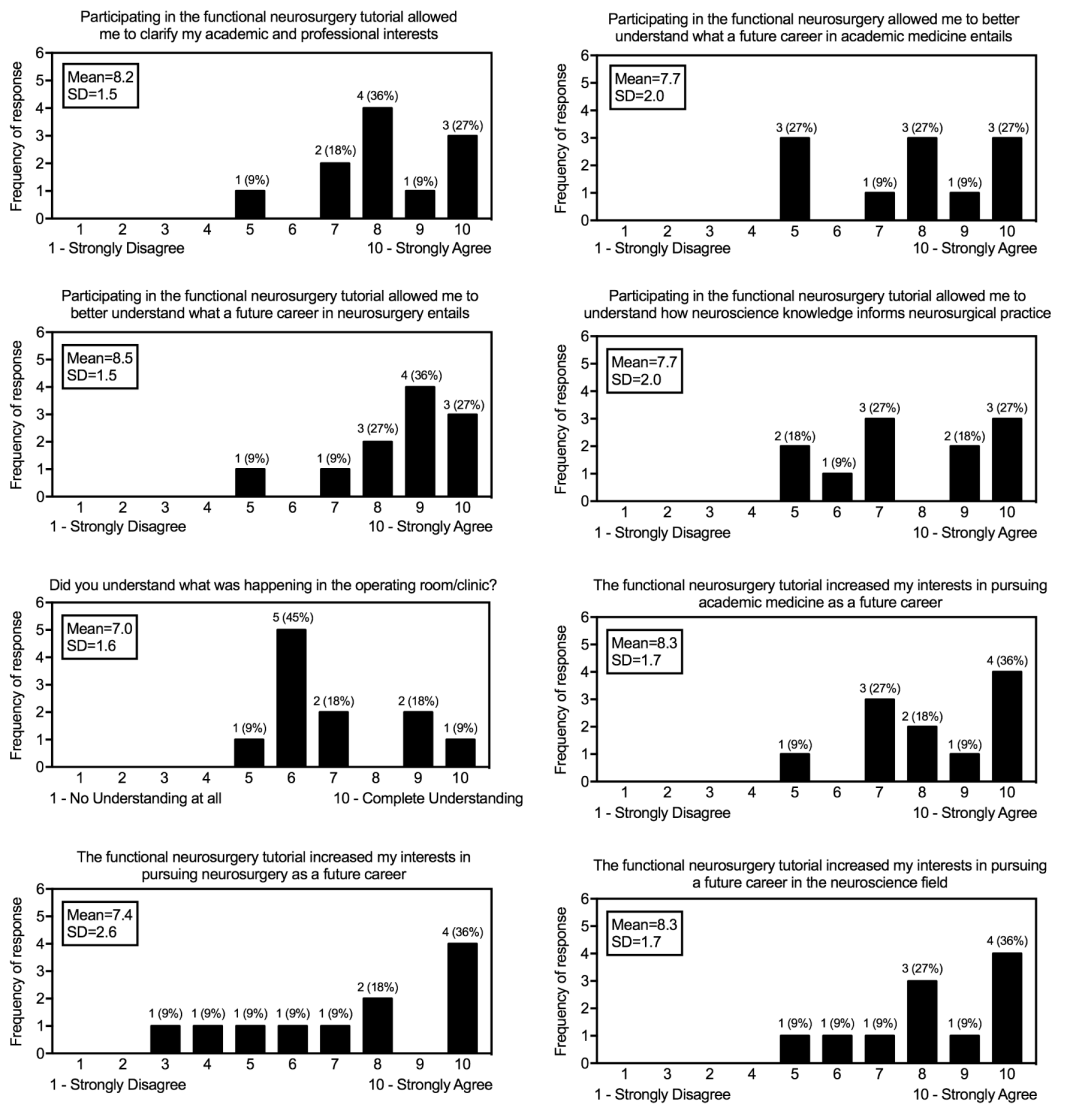
Survey results from participants in the functional neurosurgery tutorial.

the “extinction of the surgeon scientist” [27]. Indeed, a career as a neurosurgeon-scientist is uniquely challenging due to the difficulties in balancing a rigorous basic science research program with the immense clinical demands of a procedural clinical specialty. However, there are numerous examples of successful neurosurgeon-scientists, and the general consensus is that such a dual career is feasible, albeit difficult [28].

Training has been cited to be a crucial component in addressing challenges in increasing surgeon participation in basic science research [27, 29, 30]. Developing the next generation of academic neurosurgeons begins with attracting gifted students into the field [16] by providing early and sustained exposures to trainees and connecting them with successful neurosurgeon-scientists who can serve as mentors and role models [18]. Indeed, previous work suggests that shadowing programs are potentially effective in increasing student interests in pursuing careers in trauma surgery [6]. However, there exist few formal programs in surgery that combine clinical experience in the operating room with dedicated basic science research training [16]. Here, we describe and assess the educational value of a functional neurosurgery clinical shadowing and research tutorial for pre-medical trainees at the Johns Hopkins University School of Medicine Department of Neurosurgery. Program participants were exposed to all steps of conducting scholarly research in neurosurgery-related fields, including critical reading of scientific literature, generating research questions, formulating hypotheses, writing IRB research protocols, conducting experiments, analyzing data, preparing manuscripts for publication, and navigating the peer-review process in professional scientific and medical journals. Coupled with these research experiences, participants also had ample opportunities to observe neurosurgical procedures in the operating room as well as participate in clinical conferences and other formal didactic activities. Based on a survey completed by the majority of program participants, the functional neurosurgery medical tutorial increased interest in neurosurgery, neuroscience, and academic medicine as potential careers among some students by allowing direct exposure to patient clinic visits, operating room procedures, and laboratory research. Programs such as the functional neurosurgery medical tutorial, when implemented on a larger group, may be crucial for attracting trainees into the neurosurgical field and help to address concerns in the shortage of the neurosurgeon workforce in the US [17, 18, 19]. Importantly, the functional neurosurgery tutorial is unique in its integration of original scientific research with clinical experience in the neurosurgery operating rooms, allowing trainees to gain an exposure in bench-to-bedside research and appreciate how basic neuroscience discoveries can be translated into diverse avenues of clinical treatments, diagnosis, and patient outcomes. Our results further suggest that development of other surgery tutorial programs within the Johns Hopkins University as well as other academic institutions, are warranted for more definitive evaluations of the efficacy of this approach, and may be critical to continue to attract and engage students to academic medicine and surgical fields. Altogether, our work is a preliminary but important step in addressing the educational challenge of training academic neurosurgeons by providing a potential paradigm for integrating scholarly research with clinical experience in the operating room to increase interest and awareness into a career of a neurosurgeon-scientist.

## References

[1] Keswani SG, Moles CM, Morowitz M (2017). The Future of Basic Science in Academic Surgery. Ann Surg.

[2] (2018). Natl. Resid. Matching Program.

[3] (2012). Statement of the American Association of Neurological Surgeons, American Board of Neurological Surgery, Congress of Neurological Surgeons, Society of Neurological Surgeons before the Institue of Medicine On the Subject of Ensuring an Adequate Neurosurgica.

[4] Alverdy JC (2019). Surgeon as Basic Bench Scientist: A Play in Three Acts. J Surg Res.

[5] Akhigbe T, Zolnourian A, Bulters D (2017). Mentoring models in neurosurgical training: Review of literature. J Clin Neurosci.

[6] Stroh DA, Ray-Mazumder N, Norman JA, Haider AH, Stevens KA, Chi A, Rushing AP, Efron DT, Haut ER (2013). Influencing medical student education via a voluntary shadowing program for trauma and acute care surgery. JAMA Surg.

[7] Allen JG, Weiss ES, Patel ND (2009). Inspiring medical students to pursue surgical careers: outcomes from our cardiothoracic surgery research program. Ann Thorac Surg.

[8] Hernandez J, Al-Saadi S, Boyle R, Villadolid D, Ross S, Murr M, Rosemurgy A (2009). Surgeons can favorably influence career choices and goals for students interested in careers in medicine. J Am Coll Surg.

[9] Zuckerman SL, Mistry AM, Hanif R, Chambless LB, Neimat JS, Wellons JC, Mocco J, Sills AK, McGirt MJ, Thompson RC (2016). Neurosurgery Elective for Preclinical Medical Students: Early Exposure and Changing Attitudes. World Neurosurg.

[10] Hanrahan J, Burford C, Ansaripour A, Smith B, Sysum K, Rajwani KM, Huett M, Zebian B (2019). Undergraduate neurosurgical conferences - what role do they play?. Br J Neurosurg.

[11] Burford C, Hanrahan J, Ansaripour A, Smith B, Sysum K, Rajwani K, Huett M, Vergani F, Zebian B (2019). Factors Influencing Medical Student Interest in a Career in Neurosurgery. World Neurosurg.

[12] University of Washington Neurological Surgery Summer Student Program.

[13] Summer Internships for College Students, Department of Neurosurgery.

[14] Undergraduates, University of Michigan Medicine.

[15] Kamour AH, Han DY, Mannino DM, Hessler AB, Kedar S (2016). Factors that impact medical student and house-staff career interest in brain related specialties. J Neurol Sci.

[16] Suliburk JW, Kao LS, Kozar RA, Mercer DW (2008). Training future surgical scientists: realities and recommendations. Ann Surg.

[17] Gottfried ON, Rovit RL, Popp AJ, Kraus KL, Simon AS, Couldwell WT (2005). Neurosurgical workforce trends in the United States. J Neurosurg.

[18] Brown AJ, Friedman AH (2007). Challenges and opportunities for recruiting a new generation of neurosurgeons. Neurosurgery.

[19] Lunsford LD, Kassam A, Chang Y-F (2004). Survey of United States neurosurgical residency program directors. Neurosurgery.

[20] Medical Tutorials, Johns Hopkins University.

[21] Salimpour Y, Mills KA, Wei Z, Duy PQ, Anderson WS (2016). Does Transcranial Direct Current Stimulation Actually Deliver DC Stimulation: Response to Letter to the Editor. Brain Stimul.

[22] Salimpour Y, Wei Z, Duy PQ, Anderson WS (2016). Does Transcranial Direct Current Stimulation Actually Deliver DC Stimulation?. Brain Stimul.

[23] Negoita S, Duy PQ, Mahajan UV., Anderson WS (2019). Timing and prevalence of revision and removal surgeries after spinal cord stimulator implantation. J Clin Neurosci.

[24] Negoita S, Boone C, Anderson WS (2016). Directionality of Medial Prefrontal Cortex and Hippocampal Interactions Is Task-Dependent. Neurosurgery.

[25] Duy PQ, Anderson WS (2018). Two Surgeries Do Not Always Make a Right: Spinal Cord Stimulation for Failed Back Surgery Syndrome. Yale J Biol Med.

[26] Rangel SJ, Moss RL (2004). Recent trends in the funding and utilization of NIH career development awards by surgical faculty. Surgery.

[27] Kibbe MR, Velazquez OC (2017). The Extinction of the Surgeon Scientist. Ann Surg.

[28] Girgis F (2013). Feasibility of a dual neurosurgeon-scientist career in Canada: a survey study. Can J Neurol Sci.

[29] Ko CY, Whang EE, Longmire WP, McFadden DW (2000). Improving the Surgeon’s Participation in Research: Is It a Problem of Training or Priority?. J Surg Res.

[30] Jones RP, Are C, Hugh TJ, Grünhagen DJ, Xu J, Balch CM, Poston GJ (2019). Reshaping the critical role of surgeons in oncology research. Nat Rev Clin Oncol.

